# Clonality, virulence and antimicrobial resistance of enteroaggregative *Escherichia coli* from Mirzapur, Bangladesh

**DOI:** 10.1099/jmm.0.000594

**Published:** 2017-09-25

**Authors:** Marie Anne Chattaway, Michaela Day, Julia Mtwale, Emma White, James Rogers, Martin Day, David Powell, Marwa Ahmad, Ross Harris, Kaisar Ali Talukder, John Wain, Claire Jenkins, Alejandro Cravioto

**Affiliations:** ^1^​ Gastrointestinal Bacteria Reference Unit, Public Health England, London, UK; ^2^​ Antimicrobial Resistance and Healthcare Associated Infections, Public Health England, London, UK; ^3^​ Centre for Clinical Microbiology, Division of Infection and Immunity, University of College London, London, UK; ^4^​ Department of Medical Microbiology, Royal Free London NHS Foundation Trust, London, UK; ^5^​ Department of Microbiology and Infection Control, Technical University of Denmark, Denmark; ^6^​ Statistics, Modelling and Economics Department, Public Health England, London, UK; ^7^​ Centre for Food and Water Borne Diseases, International Centre for Diarrhoeal Disease Research, Bangladesh; ^8^​ Norwich Medical School, University of East Anglia, Norwich, UK

**Keywords:** enteroaggregative *E. coli*, EAEC, resistance, virulence, MLST, Bangladesh

## Abstract

**Purpose:**

This study investigates the virulence and antimicrobial resistance in association with common clonal complexes (CCs) of enteroaggregative *Escherichia coli* (EAEC) isolated from Bangladesh. The aim was to determine whether specific CCs were more likely to be associated with putative virulence genes and/or antimicrobial resistance.

**Methodology:**

The presence of 15 virulence genes (by PCR) and susceptibility to 18 antibiotics were determined for 151 EAEC isolated from cases and controls during an intestinal infectious disease study carried out between 2007–2011 in the rural setting of Mirzapur, Bangladesh (Kotloff KL, Blackwelder WC, Nasrin D, Nataro JP, Farag TH *et al.* *Clin Infect Dis* 2012;55:S232–S245). These data were then analysed in the context of previously determined serotypes and clonal complexes defined by multi-locus sequence typing.

**Results:**

Overall there was no association between the presence of virulence or antimicrobial resistance genes in isolates of EAEC from cases versus controls. However, when stratified by clonal complex (CC) one CC associated with cases harboured more virulence factors (CC40) and one CC harboured more resistance genes (CC38) than the average. There was no direct link between the virulence gene content and antibiotic resistance. Strains within a single CC had variable virulence and resistance gene content indicating independent and multiple gene acquisitions over time.

**Conclusion:**

In Bangladesh, there are multiple clonal complexes of EAEC harbouring a variety of virulence and resistance genes. The emergence of two of the most successful clones appeared to be linked to either increased virulence (CC40) or antimicrobial resistance (CC38), but increased resistance and virulence were not found in the same clonal complexes.

## Introduction

Enteroaggregative *Escherichia coli* (EAEC) have been linked to acute and persistent diarrhoea among children and adults in developing countries [1–6] where malnutrition contributes to increasing the severity of symptoms and infection can impair growth and development [7].

Strains of EAEC make a significant contribution to the burden of gastrointestinal disease in Bangladesh either as an important independent causal agent [8] or in combination with other pathogens [1, 9, 10]. In many countries, bacterial gastroenteritis is managed without recourse to antibiotics but in severe cases, high-risk patients and chronic persistent infections, specific therapy is warranted [11]. In Bangladesh, symptoms of EAEC infection are often severe or persistent and so antibiotic treatment is frequently recommended. With the exception of one report highlighting an extended spectrum beta-lactamase (ESBL)-producing strain of EAEC from a recurrent urinary tract infection [12], there is very little information published on resistance to antibiotics of EAEC in Bangladesh, or indeed globally.

The causal link of EAEC in relation to disease in the human population is not absolute [13]. Multiple studies have demonstrated the heterogeneity of EAEC with respect to both plasmid and chromosomal gene content [14, 15] and the association of single virulence factors with disease is confounded by the variation in genetic background of this group [15]. An alternative approach is to define sub-groups of EAEC, which are known to be present in multiple lineages of the *E. coli* population. Indeed multi-locus sequence typing (MLST) has been used to define sub-groups of EAEC associated, more or less, with disease [16], but few studies have analysed MLST sub-groups for virulence gene content.

There are a number of EAEC putative virulence factors, the majority of which are plasmid (pAA) borne [14, 17, 18]. The principal diagnostic target is *aggR*, which regulates the transcription of other EAEC plasmid- and chromosomally-encoded genes during the control of aggregative adherence [19, 20]. There are currently five known multiple aggregative adherence fimbriae (AAF) subunits associated with *aggR* positive EAEC: AAF/1, AAF/2, AAF/3, AAF/4 [21] and AAF/5 [22] encoded by the genes *aggA, aafA, aag3a, aag4A* and *aaf5A* respectively. Other plasmid-encoded EAEC genes include the anti-aggregative transporter (*aat*) responsible for transporting a protein called dispersin across the membrane [23], the dispersin protein encoded by *aap* [18], the enteroaggregative heat stable toxin 1 (EAST-1) encoded by *astA* [24] and the plasmid-encoded heat-liable cytotoxin encoded by *pet* [25]. Chromosomally encoded virulence factors include: the type VI secretion system on the *aggR*-activated island, *aaiC* [26]; the mucinase protein involved in colonisation encoded by *pic* [27]; the shigella enterotoxin 1 (ShET1) toxin, encoded by *set1A* and *set1B* [28]; the iron-repressible high-molecular-weight protein 2 encoded by *irp2* [29] and the locus controlling intestinal epithelial adherence and toxigenic invasion *tia* [30].

There are genetically related, clonal complexes (CCs) of EAEC with a defined ability to cause disease [15, 16]. In the Chattaway *et al.* [16] study, Bangladesh EAEC isolates were found in several of the EAEC complexes with the majority of strains falling into CC38, CC295, CC31 CC10 and CC40 but assessment of EAEC virulence genes or antibiotic resistance of these key complexes was not undertaken. Understanding pathogenic and resistant CCs of EAEC in Bangladesh can (1) help clinical management of these infections to prevent malnutrition and mortality and (2) have a baseline of data to assess that will enable further studies to assess the global impact of any major CCs. Here we report the virulence factors and antimicrobial resistance (AMR) profiles associated with these complexes. This is the first study analysing in detail EAEC isolates from the rural setting of Mirzapur, Bangladesh [31]. The aim of this study is to to seek an association between AMR, ability to cause disease and genetic background of EAEC strains from Bangladesh.

## Methods

### Bacterial strains and serotyping

One hundred and fifty-seven isolates of EAEC originally isolated between 2007–2011 from the rural setting of Mirzapur, Bangladesh using presence of the *aat* or *aaiC* gene by PCR as part of a case control study [32] were included in this study. Cases were defined as having acute onset of diarrhoea (≥3 abnormally loose stools in the previous 24 h) within 7 days of study enrolment. A control was defined as having no diarrhoea in the previous 7 days enrolled within the same community within 14 days of presentation of the index case [31].

The set comprised of 96 cases and 61 controls. Serotyping of the somatic and flagella antigens [33] was carried out on the heat stable lipopolysaccharide (LPS) (somatic or O) antigens and the flagellar (H) antigens. Strains that failed to produce LPS and could not be typed were termed ‘rough’ and those that did not react with any sera in the serotyping scheme were termed ‘O unidentifiable’ or ‘H unidentifiable’.

### Multi-locus sequencing typing and genotyping

Extracted DNA was available for 151/157 EAEC Bangladesh isolate strains from a previous study which had previously defined their multi-locus sequence types [16]. The DNA from 151 EAEC isolates was screened by PCR for the plasmid encoded virulence genes: *aat, aap, astA, aggR, affA, aafA, agg3A, agg4A, aaf5A* and *pet* and chromosomal encoded genes: *pic, Set1A, aaiC* and *irp2* (Table S1, available in the online Supplementary Material). The primers and probes for *aaf5A* were designed as part of this study FIM5_F 5′-GACTGGATTCTTCAGCTTAAATTAAG-3′, FIM_R 5′-TTCATTTGATGCTGGATTGA-3′, FIM5_P ‘GAGCCCGAGCCTGTACATAGATTTGT’. Products were amplified using the 7500 Fast Real-Time PCR System (Applied Biosystems) with amplification conditions: 95 °C for 5 min followed by 95 °C for 30 s, then 60 °C for 30 s and 72 °C for 10 s. Controls used were as follows: O42 (*aafA, aat, aggR, pic, astA, set1A, aaiC, irp2, aap, pet*), E099518 (*aag3A*), 8089 (*agg4A*), 601010 (*tia*), 900063 (*aagA*), 3036 (aaf5A). The total number of positive virulence genes was counted in each strain to give a virulence score.

Phylotyping (A, B1, B2, and D) was determined by PCR [34] for the sequence type (ST) complexes that contained 5 isolates or more.

A previous study [16] described an association of EAEC CCs with disease. Therefore, the ten main EAEC CCs in Bangladesh (CC10, 155, 165, 168, 295, 31, 38, 394, 40 and 720) were analysed for an association with virulence and resistance. Data were analysed in Stata version 13.1 (StataCorp, College Station, Texas). T-tests were used to compare means of continuous variables and the Chi-square test for categorical variables, such as associations between specific genes and the proportion of cases. Differences in virulence score according to complex were examined via multivariable linear regression.

### Antimicrobial resistance typing

The antimicrobial drug susceptibilities of all 157 EAEC isolates were determined using the agar incorporation breakpoint method described in the British Society for Antimicrobial Chemotherapy guidelines [35, 36]. The concentrations of the antibiotics used for testing were: colistin (2 mg l^−1^), gentamicin (2 mg l^−1^), amikacin (8 mg l^−1^), streptomycin (8 and 16 mg l^−1^), tobramycin (2 mg l^−1^), ertapenem (0.064 and 0.5 mg l^−1^), cefoxitin (8 mg l^−1^), ceftazidime (0.5, 1 and 2 mg l^−1^), cefotaxime (0.25, 0.5 and 1 mg l^−1^), ceftiofur (1 mg l^−1^), cefpirome (1 mg l^−1^), chloramphenicol (8 and 16 mg l^−1^), trimethoprim (2 mg l^−1^), nalidixic acid (16 mg l^−1^), ciprofloxacin (0.064 and 0.5 mg l^−1^), sulfamethoxazole (256 mg l^−1^) and tetracycline (8 mg l^−1^).

Isolates were screened by multiplex PCR for the presence of CTX-M, AmpC, TEM, SHV, VEB, PER and GES beta-lactamase genes and ESBL production was confirmed by double-disc synergy test. Group 9 CTX-M genes were identified to allele level by sequencing where possible [37]. Group 1 CTX-M alleles and their upstream genetic environments were investigated by PCR and sequencing using primers specific for ISEcp1-like and IS26-like elements [37].

## Results

### Virulence of EAEC cases and controls versus clonal complexes

Virulence gene content between cases and controls was heterogeneous (Tables S2 and S3) and none of the individual genes were significantly associated with either cases or controls. However, the average virulence score (total number of virulence genes present) was higher in cases (7.3) than controls (6.2) (Table 1) (*P*-value=0.027). The chromosomal gene *irp2* was higher in cases (70 %) than controls (58 %) but did not reach significance (*P*=0.107). There was relatively low power to detect differences in individual genes with the sample size collected in this study as only differences of 20 % or more between cases and controls would approach 80 % power. Therefore modest differences could not be ruled out (10–20 %) in the proportion of genes between cases and controls.

**Table 1.** Percentage of virulence gene in EAEC from cases and controls

	**aggR**	**aat**	**aap**	**aggA**	**aafA**	**agg3A**	**agg4A**	**aaf5A**	**astA**	**pet**	**pic**	**setA**	**irp2**	**tia**	**aaiC**	**Virulence score**
Case (93)	72 % (67)	80 % (74)	86 % (80)	14 % (13)	22 % (20)	17 % (16)	15 % (14)	18 % (17)	43 % (40)	22 .% (21)	44 % (41)	45 % (42)	70 % (66)	37 % (34)	45 % (42)	7.3
Control (58)	67 % (40)	83 % (50)	83 % (50)	17 % (10)	22 % (13)	13 % (8)	17 % (10)	13 % (8)	47 % (28)	23 % (14)	48 % (29)	40 % (24)	58 % (35)	37 % (22)	56 % (33)	6.2
Probability	0.479	0.562	0.65	0.65	0.981	0.52	0.811	0.419	0.657	0.914	0.607	0.529	0.107	0.989	0.196	0.027

Table showing the content of each gene in association with cases and controls from 151 EAEC from Bangladesh.

The virulence genes *aggR, aat, aap, AAF1-4, astA, pet, pic, setA, ipr2* and *tia* were significantly associated with the common EAEC CCs [16]. There was no statistical significant association with the common EAEC CCs for *aaf5A* and *aaiC* (Table S4). Comparison of virulence scores between EAEC CCs revealed that isolates from CC40 and CC295 harboured more virulence factors than other CCs (Table 2). Within CCs, there was no significant association between fimbrial type and whether the strain was from a case or control. ST165 strains (cases only) were positive for *agg*3A and all ST40 strains (both cases and controls) were positive for *aaf*A.

**Table 2.** Mean virulence score (and association with disease) of EAEC complexes

**Clonal complex**	**Phylogroup**	**Sample size**	**Mean of virulence score**	Association of EAEC CC with disease*
10	A	18	6.8	0.01
155	B1	8	6.6	0.2
165	A	5	4.6	0.3
168	A	5	6.8	0.2
295	B1	20	9.2	0.2
31	D	11	7.7	Associated with controls=P0.005
38	D	21	6.3	0.3
394	D	7	5.6	0.56
40	B1	10	10.4	0.03

*Taken from Chattaway *et al.* study [16].

Mean of virulence scores per EAEC Clonal complex (CC) from Bangladesh with 5 or more samples. CC40 and 295 are the most virulent in terms of average virulent gene content.

Phylogrouping showed that CCs were evenly distributed among three groups (A, B1, D) although no CC was associated with group B2. Isolates from group B1 had a higher average virulence score (8.7) than isolates from group A (6.1) and D (6.5) (Table 2) but there was no obvious association with disease from the case control data. However, analysing the groups by sequence type revealed that B1 could be further divided into three groups: CC155, 295 and 40 (Table 2). Comparison with the case control data showed that CC40 was also associated with disease (case control study *P*=0.03 [16]) and was the sequence type that harboured the most virulence factors (10.4, Table 2). Thus CC40 has been shown by two independent methods to be a virulent subtype of EAEC.

### Resistance of EAEC clonal complexes

The phenotypic antimicrobial typing results showed a high incidence of multidrug resistance (MDR) in this dataset (defined as resistant to three or more antibiotic classes). MDR was identified in 119 (75.8 %) of 157 isolates. Thirty-two (20.4 %) of isolates were resistant to the third-generation cephalosporins (ceftazidime 0.25 mg l^−1^ and cefotaxime 1 mg l^−1^) and 120 (75.9 %) were resistant to ampicillin (8 mg l^−1^). One hundred and ten (70.2 %) exhibited reduced susceptbility (0.064 mg l^−1^ ciprofloxacin) and 21 were resistant (0.5 mg l^−1^ ciprofloxacin) to the quinolones. The isolates were also resistant to streptomycin 16 mg l^−1^ (*n*=49; 31 %), trimethoprim 2 mg l^−1^ (*n*=88; 56 %) and tetracycline 8 mg l^−1^ (*n*=70; 44 %) (Table S5). Analysis of resistance gene content by CC indicated multiple drug resistance of strains within these complexes (Table 3). Of the 32 presumptive ESBL isolates, 28 had double-disk (DD) synergy and resistance was encoded by *bla*_CTX-M_ (*bla*_CTX-M-9_, *n*=17; bla_CTX-M-15_, *n*=11) which were identified as the sole mechanism explaining third-generation cephalosporin resistance.

**Table 3.** Number of resistant isolates from each complex in the dataset

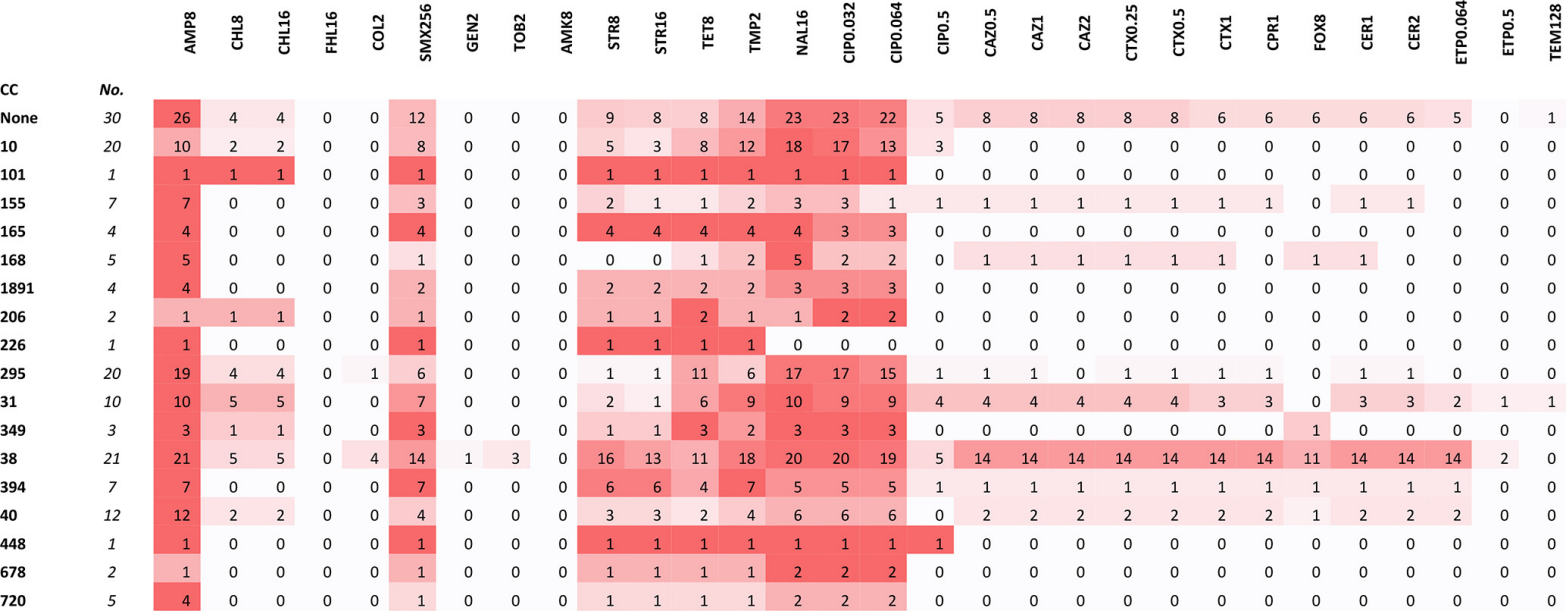

Table summarising the number of resistant isolates from each main clonal complex (CC) in the Bangladesh dataset. No=number of isolates in that complex that were resistant against the antibiotic. Colour Key=For each CC per row, the more prevalence the resistance within that complex (i.e. the greatest number of isolates per antibiotic), the darker the red.

Antibiotics: COL (colistin, 2 mg l^−1^), GEN (gentamicin, 2 mg l^−1^), AMK (amikacin, 8 mg l^−1^), STR (streptomycin, 8 and 16 mg l^−1^ respectively), TOB (tobramycin, 2 mg l^−1^), AMP (ampicillin, 8 mg l^−1^), ETP1 and ETP2 (ertapenem, 0.064 and 0.5 mg l^−1^ respectively), FOX (cefoxtin, 8 mg l^−1^), TAZ 0.5, TAZ 1 and TAZ2 (cefazidime 0.5, 1 and 2 mg l^−1^ respectively), CTX 0.25, CTX 1 and CTX 2 (cefotaxime 0.25, 0.5 and 1 mg l^−1^ respectively), CER 1 (ceftiofur, 1 mg l^−1^), CPR (cefpirome, 1 mg l^−1^) CHL1 and CHL2 (chloramphenicols, 8 and 16 mg l^−1^ respectively), TMP (trimethoprim, 2 mg l^−1^), NAL (nalidixic acid, 16 mg l^−1^), CIP 0.32, CIP 1 and CIP2 (ciprofloxacin 0.032, 0.064 and 0.5 mg l^−1^ respectively), SMX (sulfamethoxazole, 256 mg l^−1^), TET (tetracycline, 8 mg l^−^^1^).

## Discussion

This study showed that isolates of EAEC belonging to CC295 and 40 were associated with the highest virulence factor score, whereas ST165 and CC394 had the lowest virulence factor scores. This supports previous evidence from case control studies that highlighted CC40 as a potentially virulent group of EAEC [16, 38]. In previous studies, it was not always possible to correlate the presence of individual EAEC virulence factors with disease [39, 40] but this study showed an association between the presence of combined EAEC virulence factors with disease (*P*=0.027; virulence score in case versus control). While the individual virulence genes were not independently associated with the ability to cause disease (hence the non-significance association of individual gene with cases), the number of virulence genes present may increase the pathogenic potential of the strain. The heterogeneity of virulence profiles (Tables 1 and 2) suggests multiple acquisition events of different virulence genes. Evolutionary analysis of the different EAEC complexes have indicated that some clonal complexes have evolved predominantly by recombination (such as CC38) rather than mutation (such as CC10) [41]. Clonal complexes that can successfully encounter multiple recombination events can easily acquire mobile genetic elements without impact to survival of the organism. Although the functions of many of the genes described here have been published, a comprehensive understanding of all the gene functions and how they interact with other genes has yet to be fully elucidated.

We also considered the possibility that the presence of multiple virulence factors may be associated with a specific genetic background (as defined by EAEC CC). ST40 has been previously linked to a household outbreak of EAEC O111:H21 encoding the *stx*2 gene in Northern Ireland providing further evidence that CC40 has the capacity to acquire and maintain a repertoire of virulence genes [38].

The chromosomal marker *aaiC* is located on the AAI pathogenicity island that encodes a type VI secretion system regulated by the a*ggR* activator [42]. The gene *aaiC* is an important diagnostic marker for EAEC and is now used alongside *aat* or a*ggR* to detect EAEC. It has been postulated that the presence of the AAI operon may be associated with increased pathogenicity irrespective of other virulence factors [26]. However, as with previous studies [3, 14, 15, 43], this study showed no statistical association with the presence of *aaiC* in cases versus controls [3, 10, 14, 15, 43, 44].

Previous studies have highlighted other main EAEC complexes to be pathogenic including the EAEC CC38 associated with extra-intestinal infection, which had been previously shown to have an average virulence score of 9 (including extra-intestinal virulence markers) [41] as did CC10, also statistically shown to be associated with disease in adults [16] and in children [15]. Since data from this study has shown that virulence content is not directly proportional to association with disease, it is suggested that the content of known virulence genes for EAEC plays a role in the ability to cause disease but also in biological success and therefore advantageous to retain these genes as CC evolve. This may explain why the virulence scores of strains within the same complexes vary (Table 2).

CC38 (phylogroup D) is the most resistant of all of the CC in this study (Table 3). CC38 has adapted to both gastrointestinal and extra-intestinal environments [15, 16, 41]. The ability to colonise multiple niches may provide an increased opportunity to acquire resistance genes from a wide variety of bacterial species. Recombination events are a major contributory factor in the evolutionary history of CC38 [16] and may facilitate its ability to adapt to retaining multiple resistance mechanisms. CC10 is one of the largest CC in this study and an ancestral group of multiple pathotypes *of E. coli*. In CC10, mutation rather than recombination has had a higher impact on the evolution of this group [15, 41] and although the ability of CC10 to naturally evolve over time has enabled this group to evolve into multiple pathotypes of *E. coli*, this study showed that CC10 was less resistant than CC38 (Table 3). The results of this study showed that some CC are more resistant than others and that strains within a complex are not consistently resistant against the same panel of antimicrobials indicating independent acquisition of resistance mechanisms within a CC. As with the heterogeneous virulence score and there was no direct link between resistance with virulence in any of the CC.

Multidrug resistance was identified in over 75 % of the isolates including all of the main EAEC CCs (Tables 3 and S5). There is little Bangladesh EAEC resistance data available for comparison but the Talukdar *et al.* study screening *E. coli* resistance from household water supply showed 36 % of MDR of the *E. coli* strains [45]. Although the MDR EAEC strains from this study are relatively high, only ETEC and EPEC pathotypes were detected in this study, and additional studies in Bangladesh of EAEC resistance would be required for a fairer comparison. The widespread use of antibiotics has been linked with the selection of resistance mechanisms in pathogenic and non-pathogenic isolates of *E. coli* [46]. Southeast Asia has been identified as a hotspot for the emergence of MDR in both extra-intestinal bacteria and gastrointestinal pathogens [47]. Despite this fact, the level of MDR detected in the set of EAEC from Bangladesh is high, and includes a high percentage of low level quinolone resistance and ESBL-producing strains. Ciprofloxacin is often used to treat gastrointestinal infections so increasing resistance in these isolates is of concern [48]. Furthermore, it is generally accepted that gut pathogens and gut commensals act as a reservoir of resistance genes that may be acquired by extra-intestinal pathogens associated with life-threatening conditions, such as septicaemia and pneumonia [49].

In conclusion we believe that the definition of the EAEC group of bacteria includes a mixture of commensal bacteria, opportunistic pathogens and primary pathogens. Only by defining the genetic basis of virulence and biological success in these three distinct groups of EAEC will we be able to understand the association with gastrointestinal disease. This study shows that it is possible to define pathogenic and MDR CCs of EAEC and that EAEC CC40 is likely to represent a true pathogen.

## Supplementary Data

356 Supplementary File 1
