# Supplementary material for: Clonality, virulence and antimicrobial resistance of enteroaggregative Escherichia coli from Mirzapur, Bangladesh
Source: J Med Microbiol. 2017 Sep 25;66(10):1429–35. doi: 10.1099/jmm.0.000594 (PMC5845566; doi:10.1099/jmm.0.000594)
Supplement: Supplementary File 1 [file jmm-66-1429-s001.pdf]

Supplementary Table 1: Primers and probes used in this study

Target gene	Primer	Primer Sequence	Function	Reference
aat	aat_F<br>aat_R	CTG GCG AAA GAC TGT ATC AT<br>CAA TGT ATA GAA ATC CGC TGT T	Anti-aggregation protein transporter gene, Part of protein transporter system	[1]
aap	aap_F<br>aap_R	CTTTTCTGGCATCTTGGGT<br>GTAACAACCCCTTTGGAAGT	Dispersin gene	[2]
aaiC	aaiC_F<br>aaiC_R<br>aaiC_P	CATTTCACGCTTTTTCAGGAAT<br>CCTGATTTAGTTGATTCCCTACG<br>CACATACAAGACCTTCTGGAGAA	aaiC from 042 pheU island	[3]
astA	astA_F<br>astA_R	CCA TCA ACA CAG TAT ATC CGA<br>GGT CGC GAG TGA CGG CTT TGT	Enteroaggregative heat stable toxin 1 (EAST-1)	[1]
aggR	aggR_F<br>aggR_R<br>aggR_P	CCATTTATCGCAATCAGATTAA<br>CAAGCATCTACTTTTGATATTCC<br>CAGCGATACATTAAGACGCCTAAAGGA	Transcriptional activator of AAFs	[4]
aggA	aggA_F<br>aggA_R	GCT AAC GCT GCG TTA GAA AGA CC<br>GGA GTA TCA TTC TAT ATT CGC C	AAF/1 fimbrial type I	[2]
aafA	aafA_F<br>aafA_R	GAC AAC CGC AAC GCT GCG CTG<br>GAT AGC CGG TGT AAT TGA GCC	AAF/II fimbrial type II	[2]
agg3A	agg3A_F<br>agg3A_R	GTA TCA TTG CGA GTC TGG TAT TCA G<br>GGGC TGT TAT AGA GTA ACT TCC AG	AAF/III fimbrial type III	[5]
agg4A	agg4A_F<br>agg4A_R	ATA CTT TAG ATA CCC CTC ACG CAG<br>TCC ATT ATG TCA GGC TGC AA	AAF/IV fimbrial type IV	[6]
aaf5A	aaf5A_F<br>aaf5A_R<br>aaf5A_P	GACTGGATTCTTCAGCTTAAATTAAG<br>TTCATTTGATGCTGGATTGA<br>GAGCCCGAGCCTGTACATAGATTTGT	AAF/V fimbrial type V	This Study

pic	Pic_F	TTC AGC GGA AAG ACG AA	Secreted protease (146kDA), 116kDa after cleavage, multifunctional protein involved in enteric pathogenesis.	[2]
	Pic_R	TCT GCG CAT TCA TAC CA		
pet	pet_F<br>pet_R	CCGCAAATGGAGCTGCAAC<br>CGAGTTTTCCGCCGTTTTC	Plasmid encoded toxin	[7]
Irp2	Irp2_F<br>Irp2_R	AAG GAT TCG CTG TTA CCG GAC<br>TCG TCG GGC AGC GTT TCT TCT	Yersiniabactin biosynthetic gene	[8]
Set1A	Set1A_F<br>Set1A_R	TCA CGC TAC CAT CAA AGA<br>TAT CCC CCT TTG GTG GTA	Shigella enterotoxin, anti-sense strand of pic	[9]
tia	tia_F<br>tia_R	AGT GAT AGC GGA GAT GAT TG<br>CTC ACC CCG CTA TTT ATA TT	putative invasion determinant	[10]

1. Jenkins C, Tembo M, Chart H, Cheasty T, Willshaw GA, Phillips AD, et al. Detection of enteroaggregative Escherichia coli in faecal samples from patients in the community with diarrhoea. J Med Microbiol. 2006;55(Pt 11):1493-7.

2. Piva IC, Pereira AL, Ferraz LR, Silva RS, Vieira AC, Blanco JE, et al. Virulence markers of enteroaggregative Escherichia coli isolated from children and adults with diarrhea in Brasilia, Brazil. J Clin Microbiol. 2003;41(5):1827-32.

3. EU Reference Laboratory for E.coli Department of Veterinary Public Health and Food Safety. Detection of enteroaggregative *E. coli* in food by real-time PCR amplication of *aagR* and *aaiC* genes. 2013. p. 1-4.

4. Chattaway MA, Jenkins C, Ciesielczuk H, Day M, DoNascimento V, Day M, et al. Evidence of Evolving Extraintestinal Enteroaggregative Escherichia coli ST38 Clone. Emerg Infect Dis. 2014;20(11):1935-7. doi: 10.3201/eid2011.131845 [doi].

5. Bernier C, Gounon P, Le BC. Identification of an aggregative adhesion fimbria (AAF) type III-encoding operon in enteroaggregative Escherichia coli as a sensitive probe for detecting the AAF-encoding operon family. Infect Immun. 2002;70(8):4302-11.

6. Boisen N, Ruiz-Perez F, Scheutz F, Krogfelt KA, Nataro JP. Short report: high prevalence of serine protease autotransporter cytotoxins among strains of enteroaggregative Escherichia coli. Am J Trop Med Hyg. 2009;80(2):294-301. doi: 80/2/294 [pii].

7. Sheikh J, Czeczulin JR, Harrington S, Hicks S, Henderson IR, Le BC, et al. A novel dispersin protein in enteroaggregative Escherichia coli. J Clin Invest. 2002;110(9):1329-37. doi: 10.1172/JCI16172 [doi].

8. Schubert S, Rakin A, Karch H, Carniel E, Heesemann J. Prevalence of the "high-pathogenicity island" of Yersinia species among Escherichia coli strains that are pathogenic to humans. Infect Immun. 1998;66(2):480-5.

9. Huang DB, Mohamed JA, Nataro JP, DuPont HL, Jiang ZD, Okhuysen PC. Virulence characteristics and the molecular epidemiology of enteroaggregative Escherichia coli isolates from travellers to developing countries. J Med Microbiol. 2007;56(Pt 10):1386-92.

10. Jenkins C, Chart H, Willshaw GA, Cheasty T, Smith HR. Genotyping of enteroaggregative Escherichia coli and identification of target genes for the detection of both typical and atypical strains. Diagn Microbiol Infect Dis. 2006;55(1):13-9.

Isolate	Pathogen	Serotype	ST	ST Complex	Case/ Control	aggR	aat	aap	aggA	aafA	agg3A	agg4A	aaf5A	astA	pet	pic	setA	irp2	tia	aaiC	Virulence Score
900512	Case	O?:H23	157	None	1	■	■	■										1	1		6
900694	Case	O?:H24	219	None	1	■	■	■					■					1	1		7
900108	Case	O21:H8	1295	None	1		■	■						■					1	1	6
900550	Case	O128:H12	1326	None	1	■	■	■				■						1			6
1037	Case	O159:H23	1490	None	1									■							1
600965	Case	O126:H7	1891	None	1	■	■	■				■						1			6
600961	Case	O69:H38	3107	None	1							■						1			3
7071	Case	O130:H35	3670	None	1		■							■	1	1	1	1	1	1	9
601158	Case	O2:H-	3738	None	1			■			■			■						1	5
1038	Case	O25:H-	3931	None	1		■														2
7155	Case	O25:H-	3931	None	1													1			1
900442	Case	O121:H?	DLV	None	1	■	■	■		■				■	1	1	1	1		1	11
900794	Case	O?:H10	SLV	None	1	■	■	■			■	■			1			1			8
900851	Case	O125ac:H11	SLV	None	1																0
900753	Case	O166:H15	SLV	None	1	■	■	■	■			■		■				1			8
601062	Case	O2:H42	SLV	None	1			■						■					1	1	4
900553	Case	O51:H12	TLV	None	1	■	■	■		■			■	■		1		1	1		10
900245	Case	O161:H-	TLV	None	1	■	■	■			■		■	■	1	1	1	1			11
1024	Case	O130:H27	10	ST10 Cplx	1			■						■		1	1	1	1	1	7
1116	Case	O?:H33	10	ST10 Cplx	1			■				■		■	1		1	1	1	1	9
3042	Case	O3:H2	10	ST10 Cplx	0	■	■	■	■					■		1	1	1		1	9
601090	Case	O?:H33	10	ST10 Cplx	1					■						1	1	1	1	1	7
601091	Case	O?:H-	10	ST10 Cplx	1					■						1	1	1	1	1	7
601096	Case	O?:H33	10	ST10 Cplx	1											1	1	1	1	1	5
900008	Case	O?:H10	34	ST10 Cplx	1	■	■	■					■			1	1	1		1	9
600978	Case	O?:H11	48	ST10 Cplx	1		■			■								1			4
8016	Case	O?:H10	SLV	ST10 Cplx	1													1			1
1027	Case	O?:H-	SLV	ST10 Cplx	1	■	■	■							1	1	1	1		1	9
900616	Case	O113:H-	SLV	ST10 Cplx	1	■	■	■					■		1		1	1		1	9
900575	Case	O?:H10	SLV	ST10 Cplx	1	■	■	■		■				■	1	1	1	1		1	11
7040	Case	O60:H22	101	ST101 Cplx	1											1	1	1		1	4
8192	Case	O?:H19	58	ST155 Cplx	1		■	■											1		4
900098	Case	O126:H27	155	ST155 Cplx	1	■	■	■										1			5
601191	Case	O9:H21	155	ST155 Cplx	1			■						■		1	1	1		1	6
601307	Case	O9:H21	155	ST155 Cplx	1			■						■		1	1	1		1	6
900157	Case	O34:H11	223	ST155 Cplx	1		■	■				■									4
600985	Case	O77:H34	SLV	ST155 Cplx	1			■		■				■		1	1	1		1	8
900603	Case	O?:H19	165	ST165 Cplx	1	■	■	■			■										5
900820	Case	O?:H19	165	ST165 Cplx	1	■	■	■			■										5
900547	Case	O?:H19	165	ST165 Cplx	1	■	■	■			■										5
900500	Case	O128ab:H12	SLV	ST165 Cplx	1	■		■			■										4
900696	Case	O7:H4	484	ST168 Cplx	1	■	■	■				■						1			9

601235	Case	O7:H4	484	ST168 Cplx	1																5	
900732	Case	O?:H7	1891	ST1891 Cplx	1																9	
900769	Case	O69:H4	1891	ST1891 Cplx	1																4	
7207	Case	O91:H9	226	ST226 Cplx	1																9	
8080	Case	O125ac:H9	295	ST295 Cplx	1																5	
900286	Case	O?:H27	295	ST295 Cplx	1																9	
601251	Case	O?:H29	295	ST295 Cplx	1																10	
900020	Case	O?:H7	841	ST295 Cplx	1																10	
8120	Case	OR:H16	3748	ST295 Cplx	1																11	
900545	Case	O181:H16	3748	ST295 Cplx	1																12	
601110	Case	O25:H7	3748	ST295 Cplx	1																2	
7079	Case	O25:H-	SLV	ST295 Cplx	1																11	
601226	Case	O?:H27	SLV	ST295 Cplx	1																7	
900985	Case	O?:H23	130	ST31 Cplx	1																5	
900268	Case	O44:H34	130	ST31 Cplx	1																11	
900422	Case	O176:H34	130	ST31 Cplx	1																4	
901006	Case	O44:H34	130	ST31 Cplx	1																10	
600988	Case	O166:H16	130	ST31 Cplx	1																5	
600974	Case	O15:H34	SLV	ST31 Cplx	1																12	
601010	Case	O15:H23	SLV	ST31 Cplx	1																4	
600990	Case	O166:H15	349	ST349 Cplx	1																5	
7060	Case	O?:H27	38	ST38 Cplx	1																5	
900252	Case	O153:H30	38	ST38 Cplx	1																7	
900033	Case	O153:H30	38	ST38 Cplx	1																7	
900002	Case	O153:H30	38	ST38 Cplx	1																5	
900745	Case	O153:H30	38	ST38 Cplx	1																4	
601264	Case	O153:H30	38	ST38 Cplx	1																7	
601070	Case	O?:H30	38	ST38 Cplx	1																5	
900516	Case	O86:H30	38	ST38 Cplx	1																7	
601029	Case	O?:H32	38	ST38 Cplx	1																7	
900912	Case	O?:H30	315	ST38 Cplx	1																1	
900978	Case	O?:H30	315	ST38 Cplx	1																5	
900654	Case	O?:H18	SLV	ST38 Cplx	1																6	
900088	Case	O44:H40	394	ST394 Cplx	1																5	
900673	Case	O?:H18	394	ST394 Cplx	1																6	
900416	Case	O?:H18	394	ST394 Cplx	1																4	
600970	Case	O?:H18	394	ST394 Cplx	1																7	
601009	Case	O166:H15	394	ST394 Cplx	1																11	
900657	Case	O175:H28	200	ST40 Cplx	1																12	
900618	Case	O175:H7	200	ST40 Cplx	1																10	
900987	Case	O175:H31	200	ST40 Cplx	1																11	
900998	Case	O175:H1	200	ST40 Cplx	1																9	
601192	Case	O175:H1	200	ST40 Cplx	1																9	
900114	Case	O127:H11	DLV	ST40 Cplx	1																8	
601068	Case	O2:H42	SLV	ST40 Cplx	1																4	
900644	Case	O?:H10	448	ST448 Cplx	1																3	

900770	Case	O?:H23	678	ST678 Cplx	1	[redacted]	[redacted]	[redacted]			[redacted]			[redacted]		[redacted]	[redacted]	[redacted]		[redacted]	10
900063	Case	O44:H-	720	ST720 Cplx	1	[redacted]	[redacted]	[redacted]	[redacted]				[redacted]	[redacted]							6
900693	Case	O44:H23	720	ST720 Cplx	1	[redacted]	[redacted]	[redacted]	[redacted]					[redacted]							6
900883	Case	O8:H9	720	ST720 Cplx	1	[redacted]	[redacted]	[redacted]	[redacted]					[redacted]							6
601176	Case	O?:H-	720	ST720 Cplx	1	[redacted]	[redacted]	[redacted]	[redacted]					[redacted]							5
																					6.602150

Please note that strains that could not be characterised by serology are termed unidentifiable. These are represented in the table as O? or H?

Isolate	Pathogen	Serotype	ST	ST Complex	Case/ Control	aggR	aat	aap	aggA	aafA	agg3A	agg4A	aaf5A	astA	pet	pic	setA	irp2	tia	aaiC	Virulence Score
3026	Control	O?:H27	TLV	None	0	■	■	■		■			■	■	1	1	1	1	1	1	12
7064	Control	O153:H36	157	None	0	■	■	■	■												4
7092	Control	O125ac:H8	1295	None	0	■	■	■						■					1		5
7004	Control	O?:H26	2067	None	0	■	■	■		■				■	1	1	1	1	1	1	11
7067	Control	O86:H27	2166	None	0	■	■	■					■					1	1		6
601106	Control	O?:H23	2186	None	0		■	■				■								1	4
601180	Control	O?:H23	2186	None	0	■	■	■	■			■									5
7121	Control	O77:H18	DLV	None	0									■		1	1			1	4
3036	Control	O113:H-	DLV	None	0	■	■	■					■		1	1	1	1		1	9
600982	Control	O166:H15	DLV	None	0	■	■	■	■					■							5
601098	Control	O?:H45	SLV	None	0																0
601051	Control	O154:H19	10	ST10 Cplx	0		■							■		1	1	1		1	6
601197	Control	O3:H41	10	ST10 Cplx	0	■	■	■	■							1	1	1		1	8
601134	Control	O?:H10	34	ST10 Cplx	0	■	■	■		■						1	1	1		1	8
7116	Control	O?:H36	SLV	ST10 Cplx	0									■							1
7201	Control	O58:H51	SLV	ST10 Cplx	0		■							■				1			3
601035	Control	O117:H27	SLV	ST10 Cplx	0		■														1
7142	Control	O89:H-	SLV	ST10 Cplx	0									■		1	1	1	1	1	6
8225	Control	O15:H34	155	ST155 Cplx	0	■		■			■			■		1	1	1	1	1	9
7081	Control	O?:H19	165	ST165 Cplx	0	■	■	■			■										4
7172	Control	O12:H4	484	ST168 Cplx	0		■	■						■				1			6
8089	Control	O7:H4	484	ST168 Cplx	0	■	■	■				■						1		1	9
3029	Control	O12:H4	484	ST168 Cplx	0	■	■	■				■	■			1	1	1		1	6
601174	Control	O78:H7	1891	ST1891 Cplx	0		■	■				■						1			1
8098	Control	O11:H16	206	ST206 Cplx	0			■													4
600983	Control	O60:H?	206	ST206 Cplx	0											1	1	1		1	8
7058	Control	O?:H16	295	ST295 Cplx	0	■	■	■				■								1	9
8129	Control	O?:H29	295	ST295 Cplx	0	■	■	■						■	1	1		1	1	1	8
8002	Control	O9a:H10	295	ST295 Cplx	0	■	■	■					■			1	1	1		1	10
7096	Control	O?:H12	295	ST295 Cplx	0	■	■	■			■	■			1	1	1	1		1	11
7078	Control	O?:H27	295	ST295 Cplx	0	■	■	■		■		■		■		1	1	1	1	1	12
601083	Control	O?:H28	295	ST295 Cplx	0		■	■		■				■	1	1		1	1	1	8
601144	Control	O125ac:H9	295	ST295 Cplx	0	■	■	■								1	1	1	1	1	5
601188	Control	O181:H3	295	ST295 Cplx	0	■	■	■			■	■									10
7089	Control	O?:H27	3748	ST295 Cplx	0	■	■	■		■				■	1	1	1	1	1	1	10
601221	Control	O84:H27	SLV	ST295 Cplx	0	■	■	■		■				■	1	1	1	1	1	1	11
601120	Control	O130:H27	31	ST31 Cplx	0	■	■	■						■		1		1		1	5
601048	Control	O?:H23	130	ST31 Cplx	0	■	■	■			■			■		1	1	1	1	1	10
601063	Control	O15:H18	449	ST31 Cplx	0		■	■					■					1		1	5
7028	Control	O44:H34	DLV	ST31 Cplx	0	■	■	■			■								1		11

3017	Control	O166:H15	349	ST349 Cplx	0											1			1	1	5
600950	Control	O166:H15	349	ST349 Cplx	0														1	1	5
7016	Control	O153:H30	38	ST38 Cplx	0														1		10
7002	Control	O181:H36	38	ST38 Cplx	0										1	1	1	1	1	1	11
601000	Control	O?:H30	38	ST38 Cplx	0																4
601108	Control	O153:H30	38	ST38 Cplx	0														1		5
601182	Control	O?:H30	38	ST38 Cplx	0													1	1		13
601225	Control	O86:H30	38	ST38 Cplx	0													1			7
7123	Control	O21:H10	315	ST38 Cplx	0																7
8095	Control	O86:H30	SLV	ST38 Cplx	0															1	5
8130	Control	O?:H34	SLV	ST38 Cplx	0														1		6
601002	Control	O?:H41	394	ST394 Cplx	0																4
601230	Control	O44:H18	394	ST394 Cplx	0																7
601033	Control	O175:H31	200	ST40 Cplx	0										1	1	1	1		1	10
601193	Control	O175:H31	200	ST40 Cplx	0										1	1	1	1		1	11
600955	control	O175:H1	SLV	ST40 Cplx	0										1	1		1			10
601017	Control	O175:H28	SLV	ST40 Cplx	0										1	1	1	1		1	12
601155	Control	O?:H30	678	ST678 Cplx	0																7
																					05172413

Please note that strains that could not be characterised by serology are termed unidentifiable. These are represented in the table as O? or H?

Supplementary Table 4: Statistical analysis of virulence gene by associated with complexes

	aggR	aat	aap	aggA	aafA	agg3A	agg4A	aaf5A	astA	pet	pic	setA	irp2	tia	aaiC
Probability associated with complex	<0.001	<0.001	<0.001	<0.001	<0.001	<0.001	0.001	0.217	0.002	<0.001	<0.001	<0.001	<0.001	0.002	0.233

Table showing the Complex included sample sizes of 5 or more including ST10 (n=18), 155 (8), 165 (5), 168 (5), 295 (20), 31 (11), 38 (21), 394 (7) and 40 (10) complexes. Majority of virulence EAEC genes were associated with the main complexes, EAEC fimbrie type 5 and *aaiC* genes were not associated of being present with the EAEC main complexes.

Specimen ID	Pathogen	Serotype	ST	ST Cplx	R-type
3026	Control	O?:H27	TLV	None	AMP/CHL8/CHL16/TET/NAL/CIP0.032/CIP0.064
7064	Control	O153:H36	157	None	AMP/SMX/STR8/STR16/TET/TMP/NAL/CIP0.032/CIP0.064/CIP0.5
900512	Case	O?:H23	157	None	AMP/TMP/NAL/CIP0.032/CIP0.064/CAZ0.5/CAZ1/CAZ2/CTX0.25/CTX0.5/CTX1/CPR/FOX/CER1/CER2/ETP0.064
900694	Case	O?:H24	219	None	AMP/SMX/STR8/STR16/TMP/NAL/CIP0.032/CIP0.064
7092	Control	O125ac:H8	1295	None	AMP/NAL/CIP0.032/CIP0.064
900108	Case	O21:H8	1295	None	AMP/NAL/CIP0.032/CIP0.064
900550	Case	O128:H12	1326	None	AMP/SMX/TMP/NAL
1037	Case	O159:H23	1490	None	AMP/TMP/NAL/CIP0.032/CIP0.064/CIP0.5/CAZ0.5/CAZ1/CAZ2/CTX0.25/CTX0.5/CTX1/FOX
600965	Case	O126:H7	1891	None	AMP/NAL/CIP0.032/CIP0.064
7004	Control	O?:H26	2067	None	AMP/CHL8/CHL16/TET/NAL/CIP0.032/CIP0.064/FOX
7067	Control	O86:H27	2166	None	AMP/TET/NAL/CIP0.032/CIP0.064
601106	Control	O?:H23	2186	None	AMP/SMX/TMP
601180	Control	O?:H23	2186	None	AMP/CAZ0.5/CAZ1/CAZ2/CTX0.25/CTX0.5/CTX1/CPR/CER1/CER2/ETP0.064
601087	Case	O?:H45	3051	None	AMP
600961	Case	O69:H38	3107	None	AMP/SMX
7071	Case	O130:H35	3670	None	AMP/CHL8/CHL16/SMX/STR8/TET/TMP/NAL/CIP0.032/CIP0.064/CIP0.5
601158	Case	O2:H-	3738	None	AMP/CHL8/CHL16/SMX/STR8/STR16/TET/TMP/NAL/CIP0.032/CIP0.064/CAZ0.5/CAZ1/CAZ2/CTX0.25/CTX0.5/CTX1/CPR/FOX/CER1/CER2/ETP0.064
1038	Case	O25:H-	3931	None	AMP/SMX/STR8/STR16/TMP/NAL/CIP0.032/CIP0.064
7155	Case	O25:H-	3931	None	AMP/NAL/CIP0.032/CIP0.064
7121	Control	O77:H18	DLV	None	AMP/CHL8/CHL16/SMX/STR8/STR16/TET/TMP/NAL/CIP0.032/CIP0.064
3036	Control	O113:H-	DLV	None	NAL/CIP0.032/CIP0.064
900442	Case	O121:H?	DLV	None	NAL/CIP0.032/CIP0.064
600982	Control	O166:H15	DLV	None	AMP
900794	Case	O?:H10	SLV	None	AMP/SMX/STR8/STR16/TET/TMP/NAL/CIP0.064/CAZ0.5/CAZ1/CAZ2/CTX0.25/CTX0.5/CTX1/CPR/CER1/CER2
900851	Case	O125ac:H11	SLV	None	AMP/TMP/CIP0.032/CIP0.064/CAZ0.5/CAZ1/CAZ2/CTX0.25/CTX0.5/FOX
900753	Case	O166:H15	SLV	None	AMP/NAL/CIP0.032/CAZ0.5/CAZ1/CAZ2/CTX0.25/CTX0.5/CTX1/CPR/CER1/CER2/ETP0.064/TEM
601098	Control	O?:H45	SLV	None	NAL/CIP0.032/CIP0.064
601101	Case	O?:H23	SLV	None	AMP
601062	Case	O2:H42	SLV	None	AMP/SMX/TMP/NAL/CIP0.032/CIP0.064/CAZ0.5/CAZ1/CAZ2/CTX0.25/CTX0.5/CTX1/CPR/FOX/CER1/CER2/ETP0.064
900553	Case	O51:H12	TLV	None	AMP/SMX/STR8/STR16/TMP/NAL/CIP0.032/CIP0.064/CIP0.5
900245	Case	O161:H-	TLV	None	STR8/STR16/TET/NAL/CIP0.032/CIP0.064
900770	Case	O69:H4	678	None	AMP/SMX/STR8/STR16/TET/TMP/NAL/CIP0.032/CIP0.063
1024	Case	O130:H27	10	ST10 Cplx	AMP/CHL8/CHL16/SMX/STR8/STR16/TET/TMP/NAL/CIP0.032/CIP0.064/CIP0.5
1116	Case	O?:H33	10	ST10 Cplx	AMP/NAL
3042	Case	O3:H2	10	ST10 Cplx	AMP/TET/NAL/CIP0.032/CIP0.064
601051	Control	O154:H19	10	ST10 Cplx	SMX/STR8/TET/TMP/NAL/CIP0.032/CIP0.064
601197	Control	O3:H41	10	ST10 Cplx	AMP/SMX/STR8/STR16/TET/TMP/NAL/CIP0.032/CIP0.064
601090	Case	O?:H33	10	ST10 Cplx	TMP/NAL/CIP0.032
601091	Case	O?:H-	10	ST10 Cplx	TMP/NAL/CIP0.032
601096	Case	O?:H33	10	ST10 Cplx	TMP/NAL/CIP0.032
3074	Control	O56:H48	34	ST10 Cplx	NAL/CIP0.032/CIP0.064
900008	Case	O?:H10	34	ST10 Cplx	AMP/SMX/TMP/NAL/CIP0.032/CIP0.064
601134	Control	O?:H10	34	ST10 Cplx	AMP/SMX/STR8/TMP/NAL/CIP0.032/CIP0.064
600978	Case	O?:H11	48	ST10 Cplx	AMP/SMX/TET/TMP/NAL/CIP0.032/CIP0.064
8016	Case	O?:H10	SLV	ST10 Cplx	AMP/SMX/STR8/STR16/TET/TMP/NAL/CIP0.032/CIP0.064/CIP0.5
7116	Control	O?:H36	SLV	ST10 Cplx	NAL/CIP0.032/CIP0.064
7201	Control	O58:H51	SLV	ST10 Cplx	
1027	Case	O?:H-	SLV	ST10 Cplx	NAL/CIP0.032/CIP0.064
900616	Case	O113:H-	SLV	ST10 Cplx	AMP/NAL/CIP0.032
900575	Case	O?:H10	SLV	ST10 Cplx	SMX/TET/TMP/NAL/CIP0.032/CIP0.064
601035	Control	O117:H27	SLV	ST10 Cplx	
7142	Control	O89:H-	SLV	ST10 Cplx	AMP/CHL8/CHL16/TET/TMP/NAL/CIP0.032/CIP0.064/CIP0.5
7040	Case	O60:H22	101	ST101 Cplx	AMP/CHL8/CHL16/SMX/STR8/STR16/TET/TMP/NAL/CIP0.032/CIP0.064
8192	Case	O?:H19	58	ST155 Cplx	AMP/SMX/STR8
8225	Control	O15:H34	155	ST155 Cplx	AMP/SMX/TMP/NAL/CIP0.032/CIP0.064/CIP0.5/CAZ0.5/CAZ1/CAZ2/CTX0.25/CTX0.5/CTX1/CPR/CER1/CER2
900098	Case	O126:H27	155	ST155 Cplx	AMP/TET/NAL/CIP0.032
601191	Case	O9:H21	155	ST155 Cplx	AMP
601307	Case	O9:H21	155	ST155 Cplx	AMP
900157	Case	O34:H11	223	ST155 Cplx	AMP/SMX/STR8/STR16/TMP/NAL/CIP0.032
600985	Case	O77:H34	SLV	ST155 Cplx	AMP

7081	Control	O?:H19	165	ST165 Cplx	AMP/SMX/STR8/STR16/TET/TMP/NAL/CIP0.032/CIP0.064
900603	Case	O?:H19	165	ST165 Cplx	AMP/SMX/STR8/STR16/TET/TMP/NAL/CIP0.032/CIP0.064
900820	Case	O?:H19	165	ST165 Cplx	AMP/SMX/STR8/STR16/TET/TMP/NAL
900500	Case	O128ab:H12	SLV	ST165 Cplx	AMP/SMX/STR8/STR16/TET/TMP/NAL/CIP0.032/CIP0.064/CIP0.5
7172	Control	O12:H4	484	ST168 Cplx	AMP/TMP/NAL/CIP0.032/CIP0.064/CAZ0.5/CAZ1/CAZ2/CTX0.25/CTX0.5/CTX1/FOX/CER1
8089	Control	O7:H4	484	ST168 Cplx	AMP/SMX/TET/TMP/NAL
3029	Control	O12:H4	484	ST168 Cplx	AMP/NAL/CIP0.032/CIP0.064
900696	Case	O7:H4	484	ST168 Cplx	AMP/NAL
601235	Case	O7:H4	484	ST168 Cplx	AMP/NAL
900732	Case	O?:H7	1891	ST1891 Cplx	AMP/NAL/CIP0.032/CIP0.064
900769	Case	O69:H4	1891	ST1891 Cplx	AMP/SMX/STR8/STR16/TET/TMP/NAL/CIP0.032/CIP0.064
900769	Case	O69:H4	1891	ST1891 Cplx	AMP/SMX/STR8/STR16/TET/TMP/NAL/CIP0.032/CIP0.063
601174	Control	O78:H7	1891	ST1891 Cplx	AMP
8098	Control	O11:H16	206	ST206 Cplx	TET/CIP0.032/CIP0.064
600983	Control	O60:H?	206	ST206 Cplx	AMP/CHL8/CHL16/SMX/STR8/STR16/TET/TMP/NAL/CIP0.032/CIP0.064
7207	Case	O91:H9	226	ST226 Cplx	AMP/SMX/STR8/STR16/TET/TMP
8080	Case	O125ac:H9	295	ST295 Cplx	AMP/SMX/TMP/NAL/CIP0.032/CIP0.064
7058	Control	O?:H16	295	ST295 Cplx	AMP/NAL/CIP0.032/CIP0.064
8129	Control	O?:H29	295	ST295 Cplx	AMP/CHL8/CHL16/TET/NAL/CIP0.032/CIP0.064
8002	Control	O9a:H10	295	ST295 Cplx	AMP/SMX/TMP/NAL/CIP0.032/CIP0.064
7096	Control	O?:H12	295	ST295 Cplx	AMP/TET/NAL/CIP0.032/CIP0.064
7078	Control	O?:H27	295	ST295 Cplx	AMP/CHL8/CHL16/TET/NAL/CIP0.032/CIP0.064
900286	Case	O?:H27	295	ST295 Cplx	AMP/CHL8/CHL16/SMX/TET/TMP/NAL/CIP0.032/CIP0.064
601083	Control	O?:H28	295	ST295 Cplx	AMP/CHL8/CHL16/SMX/TET/TMP/NAL/CIP0.032/CIP0.064
601144	Control	O125ac:H9	295	ST295 Cplx	AMP/TET/NAL/CIP0.032/CIP0.064
601188	Control	O181:H3	295	ST295 Cplx	AMP/SMX/TET/TMP/NAL/CIP0.032/CIP0.064
601251	Case	O?:H29	295	ST295 Cplx	AMP/TET
900020	Case	O?:H7	841	ST295 Cplx	AMP/TET/NAL/CIP0.032/CIP0.064
8120	Case	OR:H16	3748	ST295 Cplx	AMP
7089	Control	O?:H27	3748	ST295 Cplx	AMP/NAL/CIP0.032
7089	Control	O?:H27	3748	ST295 Cplx	AMP/NAL/CIP0.064
900545	Case	O181:H16	3748	ST295 Cplx	AMP/NAL/CIP0.032/CIP0.064
601110	Case	O25:H7	3748	ST295 Cplx	AMP/SMX/STR8/STR16/TET/TMP/NAL/CIP0.032/CIP0.064/CIP0.5/CAZ0.5/CAZ1/CAZ2/CTX0.25/CTX0.5/CTX1/CPR/CER1/CER2
7079	Case	O25:H-	SLV	ST295 Cplx	COL/NAL/CIP0.032/CIP0.064
601221	Control	O84:H27	SLV	ST295 Cplx	AMP/TET/NAL/CIP0.032/CIP0.064
601226	Case	O?:H27	SLV	ST295 Cplx	AMP
601120	Control	O130:H27	31	ST31 Cplx	AMP/SMX/TMP/NAL/CIP0.032/CIP0.064
900268	Case	O44:H34	130	ST31 Cplx	AMP/CHL8/CHL16/STR8/STR16/TET/TMP/NAL/CIP0.032/CIP0.064/CIP0.5
900422	Case	O176:H34	130	ST31 Cplx	AMP/SMX/TET/TMP/NAL
901006	Case	O44:H34	130	ST31 Cplx	AMP/CHL8/CHL16/STR8/TET/TMP/NAL/CIP0.032/CIP0.064/CIP0.5/CAZ0.5/CAZ1/CAZ2/CTX0.25/CTX0.5/CTX1/CPR/CER1/CER2/ETP0.064
601048	Control	O?:H23	130	ST31 Cplx	AMP/SMX/TET/TMP/NAL/CIP0.032/CIP0.064
600988	Case	O166:H16	130	ST31 Cplx	AMP/SMX/TET/TMP/NAL/CIP0.032/CIP0.064
601063	Control	O15:H18	449	ST31 Cplx	AMP/CHL8/CHL16/SMX/TET/TMP/NAL/CIIP0.032/CIP0.064/CAZ0.5/CAZ1/CAZ2/CTX0.25/CTX0.5/CTX1/CPR/CER1/CER2
7028	Control	O44:H34	DLV	ST31 Cplx	AMP/NAL/CIP0.032/CIP0.064/CAZ0.5/CAZ1/CAZ2/CTX0.25/CTX1/CPR/CER1/CER2
600974	Case	O15:H34	SLV	ST31 Cplx	AMP/CHL8/CHL16/SMX/TMP/NAL/CIP0.032/CIP0.064/CIP0.5/CAZ0.5/CAZ1/CAZ2/CTX0.25/CTX0.5/ETP0.064/ETP0.5/TEM
601010	Case	O15:H23	SLV	ST31 Cplx	AMP/CHL8/CHL16/SMX/TMP/NAL/CIP0.032/CIP0.064/CIP0.5
3017	Control	O166:H15	349	ST349 Cplx	AMP/SMX/TET/NAL/CIP0.032/CIP0.064
600950	Control	O166:H15	349	ST349 Cplx	AMP/CHL8/CHL16/SMX/TET/TMP/NAL/CIP0.032/CIP0.064
600990	Case	O166:H15	349	ST349 Cplx	AMP/SMX/STR8/STR16/TET/TMP/NAL/CIP0.032/CIP0.064
7016	Control	O153:H30	38	ST38 Cplx	AMP/STR8/TMP/NAL/CIP0.032/CIP0.064/CAZ0.5/CAZ1/CAZ2/CTX0.25/CTX0.5/CTX1/CPR/FOX/CER1/CER2/ETP0.064
7002	Control	O181:H36	38	ST38 Cplx	AMP/NAL/CIP0.032
7060	Case	O?:H27	38	ST38 Cplx	AMP/CHL8/CHL16/TET/NAL/CIP0.032/CIP0.064
900252	Case	O153:H30	38	ST38 Cplx	AMP/SMX/STR8/STR16/TET/TMP/NAL/CIP0.032/CIP0.064/CIP0.5
900033	Case	O153:H30	38	ST38 Cplx	AMP/CHL8/CHL16/COL/SMX/TOB/STR8/STR16/TET/TMP/NAL/CIP0.032/CIP0.064/CIP0.5/CAZ0.5/CAZ1/CAZ2/CTX0.25/CTX0.5/CTX1/CPR/CER1/CER2/ETP0.064
900002	Case	O153:H30	38	ST38 Cplx	AMP/TMP/NAL/CIP0.032/CIP0.064/CIP0.5/CAZ0.5/CAZ1/CAZ2/CTX0.25/CTX0.5/CTX1/CPR/FOX/CER1/CER2/ETP0.064
900745	Case	O153:H30	38	ST38 Cplx	AMP/CHL8/CHL16/SMX/GEN/TOB/STR8/STR16/TET/TMP/NAL/CIP0.032/CIP0.064/CIP0.5/CAZ0.5/CAZ1/CAZ2/CTX0.25/CTX0.5/CTX1/CPR/FOX/CER1/CER2/ETP0.064
601000	Control	O?:H30	38	ST38 Cplx	AMP/STR8/TMP/NAL/CIP0.032/CIP0.064/CAZ0.5/CAZ1/CAZ2/CTX0.25/CTX0.5/CTX1/CPR/FOX/CER1/CER2/ETP0.064/ETP0.5
601108	Control	O153:H30	38	ST38 Cplx	AMP/CHL8/CHL16/COL/SMX/TOB/STR8/STR16/TET/TMP/NAL/CIP0.032/CIP0.064/CIP0.5/CAZ0.5/CAZ1/CAZ2/CTX0.25/CTX0.5/CTX1/CPR/CER1/CER2/ETP0.064
601182	Control	O?:H30	38	ST38 Cplx	AMP/COL/SMX/STR8/STR16/TMP/NAL/CIP0.032/CIP0.064/CAZ0.5/CAZ1/CAZ2/CTX0.25/CTX0.5/CTX1/CPR/FOX/CER1/CER2/ETP0.064
601264	Case	O153:H30	38	ST38 Cplx	AMP
601070	Case	O?:H30	38	ST38 Cplx	AMP/SMX/STR8/STR16/TMP/NAL/CIP0.032/CIP0.064/CAZ0.5/CAZ1/CAZ2/CTX0.25/CTX0.5/CTX1/CPR/FOX/CER1/CER2/ETP0.064

601225	Control	O86:H30	38	ST38 Cplx	AMP/SMX/STR8/STR16/TMP/NAL/CIP0.032/CIP0.064/CAZ0.5/CAZ1/CAZ2/CTX0.25/CTX0.5/CTX1/CPR/CER1/CER2/ETP0.064
900516	Case	O86:H30	38	ST38 Cplx	AMP/SMX/STR8/STR16.TET/TMP/NAL/CIP0.032/CIP0.064/CAZ0.5/CAZ1/CAZ2/CTX0.25/CTX0.5/CTX1/CPR/CER1/CER2/ETP0.064
601029	Case	O?:H32	38	ST38 Cplx	AMP/STR8/STR16/TET/TMP/NAL/CIP0.032/CIP0.064
900912	Case	O?:H30	315	ST38 Cplx	AMP/SMX/STR8/STR16/TMP/NAL/CIP0.032/CIP0.064/CAZ0.5/CAZ1/CAZ2/CTX0.25/CTX0.5/CTX1/CPR/FOX/CER1/CER2/ETP0.064
7123	Control	O21:H10	315	ST38 Cplx	AMP/SMX/TET/TMP/NAL/CIP0.032/CIP0.064
900978	Case	O?:H30	315	ST38 Cplx	AMP/CHL8/CHL16/SMX/STR8/STR16/TET/TMP/NAL/CIP0.032/CIP0.064/CAZ0.5/CAZ1/CAZ2/CTX0.25/CTX0.5/CTX1/CPR/FOX/CER1/CER2/ETP0.064
8095	Control	O86:H30	SLV	ST38 Cplx	AMP/SMX/STR8/STR16/TET/TMP/NAL/CIP0.032/CIP0.064/CAZ0.5/CAZ1/CAZ2/CTX0.25/CTX0.5/CTX1/CPR/FOX/CER1/CER2/ETP0.064
8130	Control	O?:H34	SLV	ST38 Cplx	AMP/COL/SMX/STR8/TET/TMP/NAL/CIP0.032/CIP0.064/CAZ0.5/CAZ1/CAZ2/CTX0.25/CTX0.5/CTX1/CPR/CER1/CER2/ETP0.064
900654	Case	O?:H18	SLV	ST38 Cplx	AMP/SMX/STR8/STR16/TMP/NAL/CIP0.032/CIP0.064
900088	Case	O44:H40	394	ST394 Cplx	AMP/SMX/TET/TMP/NAL/CIP0.032/CIP0.064/CIP0.5
900673	Case	O?:H18	394	ST394 Cplx	AMP/SMX/STR8/STR16/TMP
900416	Case	O?:H18	394	ST394 Cplx	AMP/SMX/STR8/STR16/TET/TMP/NAL/CIP0.064
601002	Control	O?:H41	394	ST394 Cplx	AMP/SMX/STR8/STR16/TET/TMP
601230	Control	O44:H18	394	ST394 Cplx	AMP/SMX/STR8/STR16/TMP/NAL/CIP0.032/CIP0.064/CAZ0.5/CAZ1/CAZ2/CTX0.25/CTX0.5/CTX1/CPR/CER1/CER2/ETP0.064
600970	Case	O?:H18	394	ST394 Cplx	AMP/SMX/STR8/STR16/TMP/NAL/CIP0.032/CIP0.064
601009	Case	O166:H15	394	ST394 Cplx	AMP/SMX/STR8/STR16/TET/TMP/NAL/CIP0.032/CIP0.064
900657	Case	O175:H28	200	ST40 Cplx	AMP/NAL/CIP0.032/CIP0.064/CAZ0.5/CAZ1/CAZ2/CTX0.25/CTX0.5/CTX1/CPR/CER1/CER2/ETP0.064
900618	Case	O175:H7	200	ST40 Cplx	AMP/TMP/NAL/CIP0.032/CIP0.064
900987	Case	O175:H31	200	ST40 Cplx	AMP
900998	Case	O175:H1	200	ST40 Cplx	AMP/CHL8/CHL16/SMX/STR8/STR16/TMP
601033	Control	O175:H31	200	ST40 Cplx	AMP
601193	Control	O175:H31	200	ST40 Cplx	AMP
601192	Case	O175:H1	200	ST40 Cplx	AMP/NAL/CIP0.032/CIP0.064
900114	Case	O127:H11	DLV	ST40 Cplx	AMP/NAL/CIP0.032/CIP0.064
600955	control	O175:H1	SLV	ST40 Cplx	AMP/CHL8/CHL16/SMX/STR8/STR16/TET/TMP/NAL/CIP0.032/CIP0.064
601017	Control	O175:H28	SLV	ST40 Cplx	AMP/SMX/STR8/STR16/TET/TMP
601068	Case	O2:H42	SLV	ST40 Cplx	AMP/SMX/NAL/CIP0.032/CIP0.064/CAZ0.5/CAZ1/CAZ2/CTX0.25/CTX0.5/CTX1/CPR/FOX/CER1/CER2/ETP0.064
601173	Control	O175:H31	SLV	ST40 Cplx	AMP
900644	Case	O?:H10	448	ST448 Cplx	AMP/SMX/STR8/STR16/TET/TMP/NAL/CIP0.032/CIP0.064/CIP0.5
900 770	Case	O69:H4	678	ST678 Cplx	AMP/SMX/STR8/STR16/TET/TMP/NAL/CIP0.032/CIP0.064
601155	Control	O?:H30	678	ST678 Cplx	NAL/CIP0.032/CIP0.064
900063	Case	O?:H23	720	ST720 Cplx	AMP
900693	Case	O44:H-	720	ST720 Cplx	AMP/NAL/CIP0.032/CIP0.064
900883	Case	O44:H23	720	ST720 Cplx	AMP/TET/NAL/CIP0.032/CIP0.064
601175	Case	O8:H9	720	ST720 Cplx	
601176	Case	O?:H-	720	ST720 Cplx	AMP/SMX/STR8/STR16/TMP

Please note that strains that could not be characterised by serology are termed unidentifiable. These are represented in the table as O? or H?
